# Striving to be the fittest: quantitative P2/N95 respirator fit test results among hospital staff during the COVID-19 pandemic

**DOI:** 10.1017/ash.2023.503

**Published:** 2023-12-15

**Authors:** Melanie (Meilun) Zhang, Liam Hackett, Jesse Smith, Zoe Pritchard, Matthew Casey, Caitlin Low, Paul Buntine

**Affiliations:** 1 Eastern Health Clinical School, Monash University, Melbourne, Australia; 2 Department of General Surgery, Eastern Health, Melbourne, Australia; 3 Eastern Health Emergency Medicine Program, Melbourne, Australia; 4 Genitourinary Medicine, Chalmers Centre, NHS Lothian, Scotland; 5 OHS, Emergency Management and Wellbeing, Eastern Health, Melbourne, Australia; 6 Department of Anaesthesia, Pain and Perioperative Medicine, Eastern Health, Melbourne, Australia; 7 Department of Anaesthesia, Austin Health, Melbourne, Australia

## Abstract

**Objective:**

To provide fit rates for specific P2/N95 respirators and compare these results by age, sex, clean-shaven status, and fit tester experience.

**Design:**

Exploratory audit involving secondary analysis of existing quantitative fit testing data.

**Setting:**

In response to the COVID-19 pandemic, healthcare services across Australia implemented respiratory protection protocols. This study details healthcare workers’ (HCWs) fit testing results from a large Victorian public health service.

**Participants:**

Fit-tested employees of a large tertiary public health network.

**Methods:**

Fit rates for ten individual P2/N95 respirators were calculated, and the effect of age, sex, clean-shaven status, and fit tester experience was examined via logistic regression.

**Results:**

4593 employees were included, with 97.98% successfully fitting at least one respirator. Males were found to have significantly increased odds of achieving fit success compared to females (OR 11.61 95%CI 1.60–84.10). Fit rates dropped by 4% with each 1-year age increase (OR 0.96 95%CI 0.94–0.98). Clean-shaven individuals were also more likely to achieve a fit compared to non-clean-shaved individuals (OR 79.23 95%CI 10.21–614.62). More experienced fit testers also yielded significantly higher fit rates (OR 3.95, 95%CI 2.34–6.67).

**Conclusions:**

98% of staff achieved a successful fitting of at least one respirator, with three-panel flat fold models (Industree Trident, 3M Aura 9320A+, and 3M Aura 1870+) performing the most consistently. An individual’s ability to achieve a successful fit was associated with; male sex, younger age, clean-shaven status, and fit tester experience.

## Introduction

COVID-19 is caused by severe acute respiratory syndrome coronavirus 2 (SARS-CoV-2), a highly contagious and pathogenic virus transmitted through respiratory droplets and aerosols. Healthcare workers (HCWs) are at risk of developing COVID-19 infection,^1^ especially when using inadequate or incorrect use of personal protective equipment (PPE).^1,2^ Consequently, droplet, contact, and airborne PPE precautions are often required of HCWs and others identified as working in high-risk COVID-19 exposure areas in addition to non-PPE-based precautions such as environmental cleaning and negative pressure ventilation.

Globally, P2/N95 filtering facepiece respirators (FFRs) are commonly used to meet these precautionary standards, especially where airborne routes of transmission are concerned. However, FFRs only provide satisfactory airborne protection if they properly fit the user by providing an adequate seal. Many governing bodies, including the Australian and New Zealand Standards AS/NZS 1715:2009, require HCWs who use respiratory protective equipment to undertake standardized fit testing annually, a process which has been demonstrated to improve physical protection afforded to the wearer.^2–4^ The Victorian Respiratory Protection Program (RPP) guidelines (Version 1.1) published in September 2020 currently inform how local health services should implement their own respiratory PPE protocols.^5^

Qualitative fit testing (QLFT) is a pass/fail process relying on the user’s olfactory and gustatory senses to detect aerosolized test agents. Conversely, quantitative fit testing (QNFT) utilizes a particle counter to calculate a fit factor based on the number of detected aerosols in the ambient air compared to those within the breathing zone of the FFR, thus providing an objective reading that does not rely on subjective senses. In Victoria, the recommended methodology for fit testing is QNFT due to the greater protection it provides.^5^

While all P2/N95 respirators must be demonstrated to filter at least 95% of airborne particles at the most penetrating particle size, fit factors vary between brand and model, and individual characteristics such as gender, ethnicity, facial structure, and facial hair influence results.^6,7^ Subsequently, organizations typically require a variety of FFRs to meet their needs. Due to minimal available data comparing relative fit characteristics, this is often largely influenced by price and market availability. To date, there have only been three large-scale studies published that compare the success rates of various FFRs among HCWs, one detailing results from a 2007 survey of HCWs^8^ and another in two published in 2022^9^,^10^ during the COVID-19 pandemic.

This exploratory audit of the P2/N95 respirator fit test results is from a large healthcare network in Melbourne, Australia. Our aim is to provide specific fit testing pass rates for each of the FFRs assessed on HCWs and to compare these fit rates by age, sex, clean-shaven status, and fit tester experience.

## Methods

### Type of study

Secondary analysis of existing HCW respirator fit data from Eastern Health, collected during the 2020–2021 COVID-19 P2/N95 respirator fit test program.

### Sample

Eastern Health is a tertiary public health service which comprises over 50 facilities including 3 major metropolitan hospitals in Melbourne’s east. Servicing a catchment area of over 750,000 people, Eastern Health is the second largest health provider in Victoria, with more than 10,000 employees. Participants in this present study included all fit-tested Eastern Health employees who were identified as working in high-risk patient-facing or support areas.

Fit test results were obtained over a 7-month period from the 11^th^ of February 2021 to the 10^th^ of September 2021, coinciding with the introduction of a Victorian RPP protocol which recommended a set order in which masks were to be assessed. Pre-existing fit test data for some employees from prior to these dates was also included when this involved masks not re-tested during the study period.

While the recommendation is for fit testing to be carried out on clean-shaven individuals, fit testing staff were permitted to continue with fit testing on individuals with facial hair who were insistent or booked for an afternoon session with naturally quick facial hair regrowth. Consequently, our final sample included data for individuals categorized as having stubble or a “light/close” beard. To our knowledge, no individuals with a full beard were included in the final sample.

Data on age and gender were taken from Eastern Health payroll data and matched via employee number to the fit test data.

### Fit testing

The fit test process involved a minimum of three commonly available FFRs (BYD DE2322, 3M Aura 9320A+, and Halyard 46727 regular) being tested on all subjects, with subsequent models introduced in a predefined order until a minimum of three successful respirator fits were achieved. The data collection form detailing the relevant fit test protocol is presented in Appendix 1.

Fit tests were conducted by trained fit testers using the AccuFIT 9000® PRO (AccuTec-IHS, USA) with “N95 mode” enabled. The AccuFIT 9000® PRO uses a condensation nuclei counter technique to assess the number of particles in ambient air and compares it to the particles within the breathing zone of the FFR. A fit factor of ≥100 is considered a pass.^11,12^

Daily validation checks were performed to ensure adequate particle counts and that the machine was functioning properly. Testing followed the modified Occupational Safety and Health Administration (OSHA, USA) protocol, which consists of four exercises: bending at the waist, talking, turning head side to side, and looking up and down.^11,12^

P2/N95 respirators utilized in the fit test protocol are included in Table 1, along with further details regarding respirator type and appearance. All FFRs used are listed on the Australian Register of Therapeutic Goods by the Therapeutic Goods Administration.

**Table 1.** Visual representation of facepiece respirators used in eastern health fit testing protocol

Manufacturer	Model	Respirator type	Image
BYD	DE2322	Flat fold cup	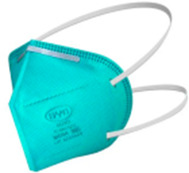
3M	Aura 1870+	Three-panel flat fold	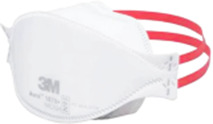
3M	Aura 9320A+	Three-panel flat fold	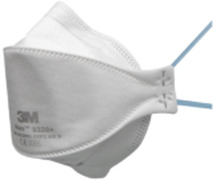
Industree	Trident	Three-panel flat fold	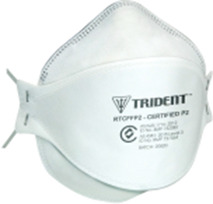
Halyard	46727 (regular)	Duckbill	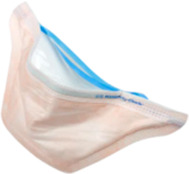
Halyard	46827 (small)	Duckbill	
BSN	72509-10 (regular)	Duckbill	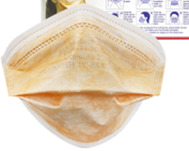
BSN	72509-09 (small)	Duckbill	
3M	1860 (regular)	Semi-rigid cup	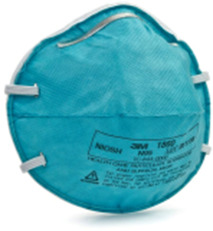
3M	1860s (small)	Semi-rigid cup	

### Statistical analysis

The characteristics of the participant cohort are presented as summary statistics with respirator pass rates presented as percentages by category. Simple and multiple logistic regression was used to compare binary pass-fail fit data for each respirator across subgroups including age, gender, clean-shaven status, and fit tester, presented as odds ratios (OR) with 95% confidence intervals. A *p*-value of <0.05 was used to denote statistical significance. Variables for inclusion in final models were selected a priori based on prior knowledge. Age, gender, clean-shaven status, and fit tester “experience” were included in final models. “Frequent” or “experienced” testers were defined as testers who had completed 100 or more individual fit tests.

All analyses were undertaken using Stata version 15.1 (StataCorp, College Station, TX).

### Ethics

This project was registered as an audit with the Eastern Health Human Research Ethics Committee (QA21-038).

## Results

### Demographics

Fit test data were available for a total of 4,593 participants, representing approximately 43% of the total employees at Eastern Health during that time.^13^ Table 2 represents the characteristics of the staff cohort. A majority of the sample were female (3416; 80.47%), and almost half of the participants were nurses (2,234; 48.67%).

**Table 2.** Characteristics of the Eastern Health fit-tested cohort^‡^

Characteristics	Mean (SD) or *n* (%)
Sex	
Male	829 (19.53%)
Female	3416 (80.47%)
Age (years)	41.03 (12.80)
Occupation	
Aboriginal and Torres Strait Islander Liaison Officer	7 (0.15%)
Aged care or disability worker	15 (0.33%)
Allied health	480 (10.46%)
Medical imaging professional	25 (0.54%)
Medical practitioner	784 (17.08%)
Midwife	174 (3.79%)
Non-clinical role	489 (10.65%)
Nurse	2234 (48.67%)
Other	301 (6.56%)
Paramedic	1 (0.02%)
Pharmacist	80 (1.74%)
Ward/Unit	
COVID/suspected COVID	233 (5.08%)
ED	560 (12.2%)
ICU	123 (2.68%)
Medical	961 (20.94%)
Non-hospital	239 (5.21%)
Other	1504 (32.77%)
Sub-acute	300 (6.54%)
Surgical	670 (14.6%)
Clean-shaven (Sex = Male)	
No	121 (17.07%)
Yes	588 (82.93%)
Testers (*N* = 84)	
Testing sessions performed	46.61 (76.6)

Note.

‡ Due to missing data points for some fields, the total for some subsections is less than 4,593.

### Fit testing pass rate outcomes

**Figure 1.**
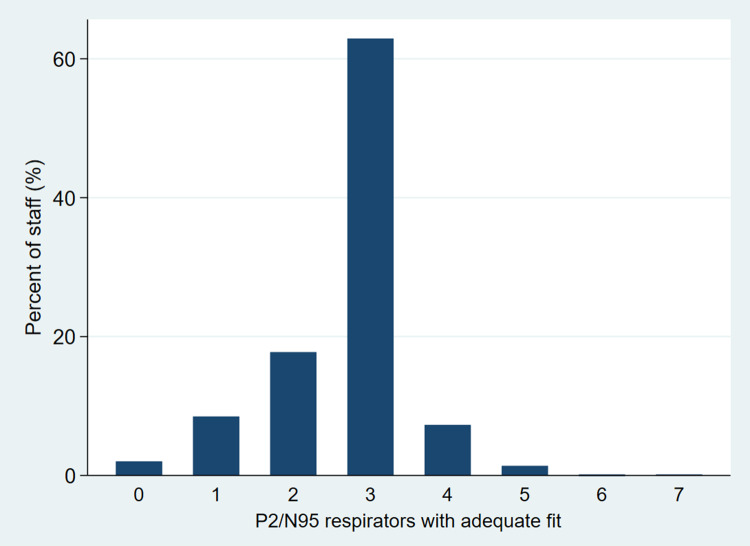
Proportion of participants by number of P2/N95 respirators with adequate fit.

Approximately 98% of employees were fitted to at least one FFR. 28% of participants were fitted to fewer than three respirators, failing to meet the target threshold of three successful respirators used to define a successful fit test. This is shown in Figure 1.

The BYD DE2322 model was the most tested FFR in our cohort, attributable to its position in the testing order and the protocolized nature of the fit testing. Despite this, it had one of the poorest fit test results, with only 30.13% of employees passing the criteria for fit.

The poorest fitting FFRs in this study were found to be the BSN 72509-09 (small) with an overall fit rate of 17.65% among the employees who tested with this mask, followed by the Halyard 46827 (small) with a 22.40% fit rate. Conversely, the Industree Trident and 3M Aura 9320A+ performed the best in our cohort, with fit rates of 86.82% and 84.44%, respectively (Table 3). As detailed in Table 3, these two FFRs had either the highest or second highest fit rate irrespective of gender or age group.

**Table 3.**
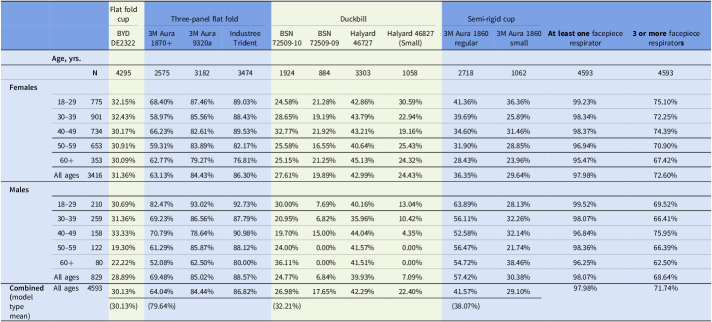
Passed fit tests by age and sex (%)

Simple and multiple logistic regressions showing the effect of s sex, age, clean-shaven status, occupation, and fit tester on fit success are shown in Tables 4 and 5.

**Table 4.** Simple logistic regression results

	BYD DE2322	3M Aura 1870+	3M Aura 9320a	Industree Trident	BSN 72509-10	BSN 72509-09	Halyard 46727	Halyard 46827 (Small)	3M Aura 1860 regular	3M Aura 1860 small	At least one facepiece respirator	3 or more facepiece respirators
Gender: Female (Ref.) Male	0.89 [0.75,1.06]	1.33* [1.06,1.67]	1.05 [0.82,1.34]	1.23 [0.93,1.62]	0.86 [0.65,1.14]	0.30** [0.14,0.62]	0.88 [0.73,1.06]	0.24*** [0.12,0.47]	2.36*** [1.95, 2.86]	1.04 [0.72,1.50]	1.05 [0.60,1.81]	0.83* [0.70,0.97]
Age (cont.):	0.99* [0.99,1.00]	0.99** [0.98,1.00]	0.98*** [0.98,0.99]	0.98*** [0.97,0.99]	1.00 [0.99,1.01]	1.00 [0.98,1.01]	1.00 [1.00,1.01]	0.99 [0.98,1.00]	0.99*** [0.98,0.99]	0.99 [0.98,1.00]	0.96*** [0.94,0.98]	0.99* [0.99,1.00]
Clean-shaven: No (Ref.) Yes	1.59* [1.04,2.43]	1.90** [1.28,2.82]	3.38*** [2.20,5.18]	1.44 [0.91,2.30]	2.29* [1.20,4.38]	7.44* [1.01,54.97]	2.65*** [1.59,4.41]	12.30* [1.68,90.06]	1.15 [0.77,1.74]	1.51 [0.73,3.10]	8.01*** [4.61,13.91]	2.42*** [1.74,3.37]
Tester: Infrequent (Ref) Frequent	1.16* [1.00,1.34]	1.91*** [1.61,2.26]	1.13 [0.91,1.40]	2.12*** [1.72,2.62]	0.92 [0.74,1.14]	0.92 [0.63,1.34]	1.22* [1.04,1.43]	0.81 [0.59,1.10]	0.83* [0.71,0.98]	0.76 [0.57,1.00]	3.79*** [2.36,6.08]	1.36*** [1.18,1.56]

Note. Presented as odds ratios (OR) and 95% confidence intervals. * *P* < 0.05 ** *P* < 0.01 *** *P* < 0.001.

**Table 5.** Multiple logistic regression results†

	BYD DE2322	3M Aura 1870+	3M Aura 9320a	Industree Trident	BSN 72509-10	BSN 72509-09	Halyard 46727	Halyard 46827 (Small)	3M Aura 1860 regular	3M Aura 1860 small	At least one mask	3 or more masks
Gender: Female (Ref.) Male	0.91 [0.74,1.12]	1.72*** [1.30,2.27]	1.52* [1.07,2.17]	1.37 [0.96,1.95]	1.02 [0.74,1.40]	0.36* [0.15,0.87]	1.02 [0.81,1.29]	0.22** [0.09,0.56]	2.40*** [1.92,3.00]	1.55 [1.00,2.42]	11.61* [1.60,84.10]	0.91 [0.74,1.11]
Age (cont.):	0.99* [0.99,1.00]	0.99 [0.99,1.00]	0.98*** [0.97,0.99]	0.98*** [0.97,0.99]	1.00 [0.99,1.01]	0.99 [0.98,1.01]	1.00 [1.00,1.01]	0.99 [0.98,1.00]	0.98*** [0.98,0.99]	0.99 [0.98,1.00]	0.96*** [0.94,0.98]	0.99* [0.99,1.00]
Clean-shaven: No (Ref.) Yes	1.27 [0.78,2.07]	2.85*** [1.70,4.78]	4.96*** [2.84,8.68]	2.30** [1.27,4.16]	2.41* [1.08,5.36]	2.11 [0.24,18.53]	2.78*** [1.51,5.09]	2.15 [0.24,19.26]	1.92** [1.17,3.16]	1.65 [0.71,3.81]	79.23*** [10.21,614.62]	2.15*** [1.43,3.23]
Tester: Infrequent (ref.) Frequent	1.21* [1.04,1.41]	1.91*** [1.59,2.29]	1.20 [0.95,1.51]	2.06*** [1.64,2.58]	0.88 [0.70,1.10]	1.08 [0.73,1.60]	1.21* [1.02,1.43]	0.83 [0.60,1.16]	0.82* [0.68,0.97]	0.70* [0.52,0.95]	3.95*** [2.34,6.67]	1.32*** [1.14,1.53]

Note.

† Adjusted for age, gender, clean-shaven status, and fit tester. Presented as odds ratios and 95% confidence intervals. * *P* < 0.05 ** *P* < 0.01 *** *P* < 0.001.

### Fit testing outcomes and gender

The odds of a successful fit test significantly differed between gender for multiple respirators. After adjusting for age and clean-shaven status, males were more likely than females to achieve fit with four of the respirators: 3M 1860 (regular), 3M Aura 1870+, the 3M Aura 9320A+, and the Industree Trident. On the contrary, males were less likely than females to fit the BSN 72509-09 (small) and Halyard 46827 (small) (OR 0.36 95%CI 0.15–0.87; OR 0.22 95%CI 0.09–0.56). These results are shown in Table 5.

### Fit testing outcomes and age

Over 99% of individuals in the 18–29 age group were able to achieve at least one successful respirator fit with a statistically significant inverse relationship between increasing age and fit success such that the adjusted odds of fitting at least one respirator dropping by 4% for each 1-year increase in age (Table 5).

### Fit testing outcomes and clean-shaven status

Clean-shaven employees were more likely to achieve fit with all FFRs tested, although this was not always statistically significant. The adjusted odds of fitting at least one respirator was greater than 70-fold for clean-shaven individuals when compared to non-clean-shaven individuals (OR 79.23 95%CI 10.21–614.62).

### Fit testing outcomes and fit tester

Our results suggest that fit testers may have some role in success rates of respirator fit, with frequently utilized testers at statistically significant increased odds in achieving at least one respirator fit when compared to less experienced fit testers (OR 3.95, 95%CI 2.34–6.67).

## Discussion

In this large study of employees at a single health network in Australia, approximately 98% of the participants achieved at least one successful respirator fit from the ten different P2/N95 respirators provided. Fit rates were inversely proportional to increasing age, with males generally better suited to the available tested respirators. Clean-shaven individuals were significantly more likely to achieve sufficient respirator seal than non-clean-shaven individuals and an association between fit tester experience and improved fit success was observed.

The results of this study are consistent with those published from previous Australian studies. Ng et al found a 96% fit rate using three-panel flat fold respirators,^10^ while Milosevic et al compared fit rates of eight FFRs and found a successful fit rate of 93%.^9^ A study by Wilkinson et al also saw a fit success rate of 83% when testing from a pool of five FFR models.^8^

Our findings indicate that the three respirators with the highest fit rates in our cohort were the three-panel flat fold respirators: Industree Trident (86.82%), 3M Aura 9320A+ (84.44%), and 3M Aura 1870+ (64.04%). These findings are echoed by other studies^8,14–16^ with three-panel respirators yielding superior fit rates compared with other respirator types.^10^ A Taiwanese study by Lin and Chen suggested that three-panel flat fold FFRs allowed for the greatest flexibility to fit various facial contours, particularly when engaging in various physical movements,^15^ which is mirrored by a French study that found semi-rigid cup models to be too firm to sufficiently adjust to the intricacies in facial characteristics.^17^ Shin et al also found the three-panel flat fold FFRs had the lowest reduction in respiratory protection when performing aerosol-generating procedures such as chest compressions.^18^ However, these findings are not universal. Milosevic et al found that semi-rigid cup models had greater success rates compared to flat fold styles,^9^ possibly due to differences in fit test protocol, as the study did not follow a predetermined order of respirators during fit testing, leading to a smaller sample size of flat fold models (3.87%) tested and the inclusion of only one three-panel flat fold model (3M Aura 1870+). Additional variables such as ethnicity and facial morphology could also possibly explain this variation in findings.

Both duckbill models were the poorest performing FFRs in the present study, with the BSN 72509-09 (small) producing a fit rate of 17.65%, followed by the Halyard 46827 (small) (22.40%). This is consistent with previously published studies in which duckbills were also found to be more likely to fail compared to their more rigid counterparts.^9,10,17,19–21^

When evaluating data on US workers to inform respirator design, males and females were found to have significantly different facial anthropometric features which can affect FFR seal.^22^ The present study noted that males were at significantly increased odds (almost 12-fold) to achieve at least one FFR fit when compared to females. This was a similar finding among other QNFT studies,^16,23–26^ including Ascott et al, who found significantly higher failure rates in females (18.2%) compared to males (9.2%),^24^ and Carvalho et al, who found that a higher proportion of females than males required multiple attempts to achieve a successful fit.^26^ Other studies found no difference between sexes,^8,27–29^ although some of these utilized sample sizes of <50.^27,29^ Milosevic et al was the sole study which found females more readily fitted compared to their male counterparts overall, but this is possibly attributable to increased testing of respirators that were more suited to the females in their cohort.^9^ Regarding specific models, males were significantly more likely than females to achieve fit in the three-panel flat fold FFRs, whereas females were more likely to achieve a seal in two of the three smaller models available—the Halyard 46827 and BSN 725909-09. It is possible that females are more likely to fit smaller respirators due to their smaller facial anthropometric dimensions.^15,22^ Despite these results, these two FFRs were still the least successful respirators tested among females in our cohort.

In a 2010 US workforce study of facial anthropometric data, Zhuang et al found that facial characteristics differed significantly among different age groups.^22^ In our study, adjusting for sex and clean-shaven status, younger staff members were significantly more likely to attain a sufficient fit for 5 of the tested FFRs compared to their older counterparts. This finding was consistent with observations made by Milosevic et al,^9^ potentially suggesting that younger HCWs are more compatible in terms of achieving a tight seal when donning present-day FFRs due to specific facial dimensions. Apart from McMahon et al, which found female HCW <40 years were less likely to achieve fit success,^23^ other QNFT studies did not appear to provide commentary on this phenomenon. Further research is required into whether other factors regarding age affect an individual’s fit, such as increased elastin and collagen composition in younger populations.^30^

Unsurprisingly, clean-shaven status significantly affected the ability of individuals to attain a successful FFR fit. This is consistent with previous literature which found facial hair to reduce fit factors by 17-fold.^31^ Accordingly, Australian Standards and NIOSH guidelines require all individuals undergoing fit testing to be cleanly shaven, to achieve best possible fit results.^4,5,12^ However, a recent observational audit in Australia found that 45% of male HCWs were not clean-shaven when utilizing N95/P2 respirators.^32^ Moreover, 17% of the male employees in our sample for which these data were collected were also classified as not being clean-shaven, suggesting that adherence to this recommendation remains poor.

A decision was made to evaluate fit testers involved in our study, revealing significantly increased odds of achieving a FFR fit if the individual was tested by a “frequent” fit tester as opposed to an “infrequent” fit tester. In a 2010 study, Wilkinson et al described a similar phenomenon attributed to experience, although fit testers were able to select respirators based on previous experience.^8^ While the predetermined FFR testing order utilised at our health network precluded testers from selecting respirators, the authors of this study suggest that the ability of a frequent tester to more clearly instruct and guide HCWs to don respirators correctly and perform test related tasks, as well as to promptly identify any incidents where this process had not occurred appropriately, may have contributed to this observed difference.

To our knowledge, this is one of the largest Australian QNFT studies comparing various models of P2/N95 respirators. Our data adds weight to the contention that age, sex, and clean-shaven status are important considerations when stocking appropriate FFRs in a healthcare setting, and sheds further light on how experience of the fit tester may affect these fit test results. Over the past 3 years, the COVID-19 pandemic has ushered in a period of great demand for respirators for our frontline workers, with supply chains heavily strained. Combined with the rise of reported adverse effects such as skin irritation and pressure sores leading to potential poor compliance with certain respirators,^33,34^ further research is indicated regarding N95 respirator availability, as well as staff compliance and satisfaction with the fit-tested respirators.

Our study has some limitations. As it was a secondary analysis of existing respirator fit data, it was unable to capture information regarding ethnicity, BMI, and participant facial anthropometric dimensions. Given reduced success rates in Asian females shown in previous literature,^8,16,25,26^ data comparing the differences in ethnic makeup and how this affected respirator fit would have been of interest. There was also a lack of data regarding non-binary participants, as this information was not recorded.

Another limitation was due to the nature of the QNFT protocol used. According to the Victorian RPP,^5^ the predefined order of respirators was dictated by the supply of respirators across Victoria during the peak of the pandemic. It is likely that this, coupled with a recommendation to cease the fit test session after 3 successful fit tests were achieved, may have affected success rates in FFRs lower in the testing order, as they were tested less (yielding a smaller sample size) and potentially on individuals with facial anthropometric dimensions that were more difficult to achieve as suitable seal in. On the other hand, the advantage of this predetermined method over a tester-guided system allowed for less confounding by the fit tester or staff preference and ensured the top three respirators were tested on all participants.

Importantly, applicability of our findings should be noted to be limited to the ten respirators involved in our testing protocol and may not be generalizable to all other circumstances due to differing respirator access and stock. Lastly, despite the QNFT protocol being a more reliable form of fit testing,^5^ this does not ensure a perfect seal each time. Sufficient respirator seal is heavily reliant on user fit checking with each application of the respirator,^5^ not just by passing a fit test alone.

In conclusion, 98% of staff achieved a successful fitting of at least one respirator, with three-panel flat fold models (Industree Trident, 3M Aura 9320A+, and 3M Aura 1870+) performing the most consistently. This was significantly affected by younger age, male sex, clean-shaven status, and experience of the fit tester which improved fit rates across majority of the tested respirators. Further research is still required to investigate FFR availability, alongside staff compliance and satisfaction with usage in the clinical setting. Ethnicity is another factor which likely heavily influences respirator fit that has not been extensively explored. Our findings support maintaining existing requirements for workers to remain clean-shaven in high-risk settings where FFRs are required and underscore the importance for respirator manufacturers to test their designs on a variety of people. We hope that the results of this study can better inform governing bodies on a global scale regarding the most appropriate respirators to order and stockpile in order to successfully protect frontline workers in the face of infectious disease.

## Supporting information

Zhang et al. supplementary material Zhang et al. supplementary material

## Supplementary material

For supplementary material accompanying this paper visit http://doi.org/10.1017/ash.2023.503. click here to view supplementary material
