# Supplementary material for: Striving to be the fittest: quantitative P2/N95 respirator fit test results among hospital staff during the COVID-19 pandemic
Source: Antimicrob Steward Healthc Epidemiol. 2023 Dec 15;3(1):e233. doi: 10.1017/ash.2023.503 (PMC10753470; doi:10.1017/ash.2023.503)
Supplement: Zhang et al. supplementary material [file S2732494X2300503Xsup001.docx]

**Appendix 1: Fit test data collection form**

| **1.** Was the staff member clean shaven? Yes      No, stubble/light/close beard        No, full beard |
| --- |

***NOTE:** The 3M Aura 1870+ are only to be used for fit testing (not clinical use)

Respirators should be tested in the order below as those listed first are in higher supply (or have a higher potential of supply) across the state.

The fit test should stop once the individual has achieved a pass on **three different models** (not including the 3M Aura 1870+).

In cases where it is obvious that a respirator will not fit the individual (e.g. it falls off the face), commence the test and allow the mask to fail so that it will be recorded as a failed fit test.

| **Order** | **Mask** | **Seal Achieved?**  = Yes = No | **Fit Achieved?**  = Yes = No | **Fit Factor and Comments** | **Fit Tester Initials** |
| --- | --- | --- | --- | --- | --- |
| 1. | BYD DE2322 |  |  |  |  |
| 2. | 3M Aura 1870+* |  |  |  |  |
| 3. | 3M Aura 9320A+ |  |  |  |  |
| 4. | Halyard 46727 (Regular) |  |  |  |  |
| 5. | Industree Trident P2 |  |  |  |  |
| 6. | 3M 1860 (Regular) |  |  |  |  |
| 7. | BSN 72509-10 (Regular) |  |  |  |  |
| 8. | Halyard 46827 (Small) |  |  |  |  |
| 9. | 3M 1860s (Small) |  |  |  |  |
| 10. | BSN 72509-09 (Small) |  |  |  |  |

 Last updated: 01_Eastern Health Consent - Fit Testing (updated based on DHHS changes)_20210503
